# PATHWAYS OF IMPROVING SOCIAL CAPABILITY OF OLDER ADULTS WITH FUNCTIONAL LIMITATIONS: FINDINGS FROM MIXED METHODS

**DOI:** 10.1093/geroni/igac059.2924

**Published:** 2022-12-20

**Authors:** Yalu Zhang, Yiyi Xiong, Gong Chen

**Affiliations:** Peking University, Beijing, Beijing, China (People’s Republic); Peking University, Beijing, Beijing, China (People’s Republic); Peking University, Beijing, Beijing, China (People’s Republic)

## Abstract

**Objectives:**

Social capabilities are the opportunities to realize people’s potential. Despite an established positive link between health status and social capabilities among older adults, the relationship mechanisms are understudied.

**Methods:**

Using the China Longitudinal Aging Social Survey data 2014–2018, this paper examined the possible mediating role of community participation through which older adults (aged 60 and above) in China with functional limitations improved or maintained their social capabilities. By conducting the 12 in-depth interviews, this study explores how community participation altered the negative associations between functional limitations and social capabilities among Chinese older adults.

**Results:**

Findings from the quantitative study show that both physical (-.136, p < 0.001) and cognitive (-.149, p < 0.001) functional limitations showed consistent and negative effects on the social capabilities of older adults, and the effects varied between males and females. The mediation analysis results show that community participation accounted for a substantial proportion of the impact of functional limitations (36.33%) on social capabilities. However, functional limitations still had strong, negative direct effects of their own. Findings from the qualitative narrative synthesis show that peer companionship, regular physical activities, and reduced digital obstacles to accessing online social media during social participation are the self-perceived driving force in enhancing their sense of security, freedom of expression, and sense of social cohesion. Discussion: Findings from this study highlight the need for more social policies and services to encourage community participation among older adults.

